# Paclitaxel-Induced Pneumonitis in Trinidad: A Case Report

**DOI:** 10.7759/cureus.26613

**Published:** 2022-07-06

**Authors:** Nishtha Mohan, Dominic Dalip, Fidel S Rampersad, Shiva Jaggernauth

**Affiliations:** 1 Internal Medicine, The University of the West Indies, Saint Augustine, TTO; 2 Internal Medicine, Southern Medical Clinic, San Fernando, TTO; 3 Medicine, Seattle Science Foundation, Seattle, USA; 4 Radiology, The University of the West Indies, Port of Spain, TTO; 5 Respiratory Medicine, Apley Medical Ltd, San Fernando, TTO; 6 Pulmonary Medicine, Southern Medical Clinic, San Fernando, TTO

**Keywords:** hypersensitivity, drug-induced, breast cancer, pneumonitis, paclitaxel

## Abstract

Paclitaxel-induced pneumonitis (PIP) is an immune-mediated disease resulting from a delayed hypersensitivity reaction (type IV) to paclitaxel, an anti-microtubule chemotherapeutic drug commonly used to treat breast cancer in both neoadjuvant and adjuvant settings. PIP is diagnosed by exclusion utilizing laboratory work-up, imaging, biopsy studies, and results of antibiotic therapy because there is no single diagnostic test. Ground-glass opacifications on CT, coupled with minimal restrictive disturbance with decreased diffusion on pulmonary function tests (PFTs), negative bronchoalveolar lavage (BAL), and bronchoscopy cultures, may assist physicians in diagnosing paclitaxel-induced pneumonitis. In this report, we describe a case of PIP present in Trinidad, West Indies, which has not been described previously in this region.

## Introduction

Paclitaxel-induced pneumonitis (PIP) is an immune-mediated disease resulting from a delayed hypersensitivity reaction (type IV) to paclitaxel, an anti-microtubule chemotherapeutic drug of the taxane class, commonly used to treat breast cancer in both neoadjuvant and adjuvant settings. Positive leukocyte migration inhibition test to paclitaxel in lymphocytes extracted during bronchoalveolar lavage of affected patients further suggests that PIP is a delayed hypersensitivity reaction [1]. Additionally, patients with PIP were noted to have a significant elevation of cyclooxygenase-2 (COX-2) protein [2]. COX-2 is a pro-inflammatory mediator that may cause lung injury by activating a cascade of inflammatory reactions, which might be the mechanism of paclitaxel-induced lung injuries [2]. PIP is a very rare, poorly characterized, and potentially life-threatening complication of paclitaxel therapy with an estimated incidence of 0.73 - 12% [1,3]. Possible risk factors of PIP include pre-existing interstitial lung disease (ILD), 12-cycle vs. 4-cycle dosing regimen, and tumor type (presuming lung cancer patients may have lower pulmonary reserves) [1,4]. PIP is diagnosed by exclusion of other causes of respiratory distress because there are no set diagnostic criteria. The option of resuming taxane chemotherapy following clinical recovery, hereafter referred to as ‘rechallenge’, has not been well documented thus far. Therefore, there is a great need for further research in this regard because the cessation of taxane chemotherapy disrupts curatively intended treatment for breast cancer patients, particularly those at high risk of developing metastatic disease [4]. Upon review of the literature, there were no published case reports on PIP in Trinidad and Tobago. We describe a case of PIP presented at the Apley Medical Clinic, Trinidad, West Indies, and the outcome of subsequent taxane rechallenge. 

## Case presentation

A 56-year-old female presented with a persistent cough of four weeks duration that worsened over time. The cough was productive of clear sputum and occurred mostly during the daytime. Following coughing fits, the patient experienced dyspnea on exertion and excessive sweating. She denied chest tightness, palpitations, and lightheadedness. The patient was a non-smoker and did not use recreational drugs. Given her history of asthma, Ventolin was initially used by the patient, which brought no relief. Additionally, she was being treated with adjuvant chemotherapy for stage 3 breast cancer and had received four of the twelve planned cycles of paclitaxel which followed four cycles of dose-dense doxorubicin and cyclophosphamide.

At the time of initial evaluation, vital signs showed oxygen saturation of 99% on room air, heart rate of 109 bpm, blood pressure 150/80 mmHg, and a temperature of 36.7ºC (98.0 F). Physical examination revealed clear lungs on auscultation with no crepitus or rhonchi. Polymerase chain reaction (PCR) testing for COVID-19 was negative. The differential diagnoses included gastroesophageal reflux disease (GERD), postnasal drip, pulmonary hypertension, pulmonary embolism, and drug-induced pneumonitis. She was started on medication pantoprazole, montelukast, fluticasone furoate/vilanterol inhaled, mometasone nasal spray, Tuscosed Linctus, and albuterol inhaled. Her oncologist prescribed a three-day course of azithromycin due to concern for infection, but her symptoms did not resolve.

**Figure 1:**
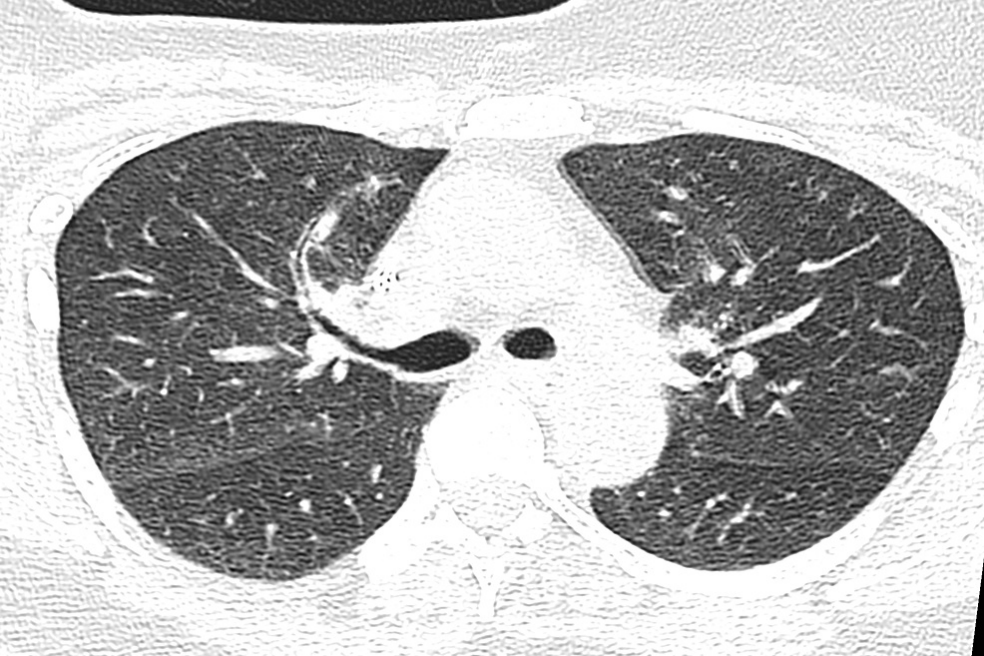
Ground-glass opacities seen in mid-zones and adjacent to the oblique and horizontal fissures

Following a week of treatment, her condition deteriorated with complaints of more frequent coughing fits. Laboratory workup revealed neutrophilia (7.6 x 109/L), negative troponin, negative myoglobin, negative autoimmune screen, and elevated d-dimer (1.42ug/mL). ECG and echocardiogram (ECHO) findings were unremarkable except for sinus tachycardia. CT pulmonary angiogram (CTPA) was negative for pulmonary embolism but revealed upper lobe mild subpleural reticular changes and mild mid-zone central ground-glass opacifications (Figure 1). Furthermore, pulmonary function tests (PFTs) demonstrated mild restrictive ventilatory defect and severe gas transfer defect (DLCO (Hb) = 11.74 mL/min/mmHg; 50% predicted) and normal DLCO/VA with no significant reversibility (Table 1). Therefore, based on the combination of clinical presentation and radiological findings, the relationship between exposure to paclitaxel and onset of respiratory distress as well as the exclusion of other causes of respiratory distress, a diagnosis of drug-induced pneumonitis secondary to paclitaxel was made. The interventions employed were immediate cessation of Paclitaxel treatment and initiation of high dose dexamethasone taper (8 mg for three days, 4 mg for three days, 2 mg for three days, 1 mg for three days).

**Table 1:** Pulmonary function test results obtained following the fourth cycle of paclitaxel, subsequent paclitaxel cessation and steroid treatment, and the first cycle of docetaxel rechallenge Pred - predicted; FVC - forced vital capacity; FEV - forced expiratory volume; DLCO - diffusion capacity of lung for carbon monoxide; VA - alveolar ventilation; TLC - total lung capacity; RV - residual volume

Parameter	Units	Post paclitaxel (Cycle 4/12)	Post paclitaxel cessation and steroid treatment	Post docetaxel (cycle 1/3) rechallenge	Pred value post docetaxel (cycle 1/3) rechallenge
Spirometry					
FVC	L	1.92	2.06	1.90	3.06
FEV_1_	L	1.42	1.48	1.36	2.47
FEV_1_/FVC	%	74	72	72	80
Diffusing capacity					
DLCO (Hb)	mL/min/mmHg	11.74	12.80	11.84	21.59
DLCO/VA	mL/min/mmHg/L	4.13	3.91	4.04	3.97
Lung volumes (He)					
TLC	L	3.28	3.43	3.09	4.92
RV	L	1.36	1.37	1.19	1.86
RV/TLC	%	41	40	39	38

Following steroid therapy, the patient reported that the cough was completely resolved. Chest X-ray and PFT test was repeated, which revealed clear lung fields and a persistent mild restrictive ventilatory defect with an improved moderate gas transfer defect (DLCO (Hb) = 12.80 mL/min/mmHg; 60% predicted), respectively (Table 1). She was deemed clinically recovering and thus was scheduled to have a rechallenge with another taxane drug, docetaxel, with careful monitoring.

Two weeks after she received the first of the three planned cycles of docetaxel, there was no recurrence of respiratory symptoms; however, PFT showed moderate restrictive ventilatory defect with severe gas transfer defect (DLCO (Hb) = 11.84 mL/min/mmHg; 55% predicted) and normal DLCO/VA (Table 1). In the absence of symptoms, she was cleared to continue with the remaining two cycles of docetaxel. She experienced a successful outcome with docetaxel rechallenge following PIP.

## Discussion

The onset of clinical manifestations associated with PIP is variable, with a range of two to 20 days from the first cycle of paclitaxel to the development of symptoms [1]. PIP is characterized by non-specific symptoms such as dry or productive cough, fever, and dyspnea; therefore, it is imperative to rule out other causes of respiratory failure, including pneumonia, cardiogenic pulmonary edema, and diffuse alveolar hemorrhage [5].

PIP is diagnosed by exclusion utilizing laboratory workup, imaging, biopsy studies, and results of antibiotic therapy because there is no one single diagnostic test [4]. Ground-glass opacifications on CT coupled with minimal restrictive disturbance with decreased diffusion on PFTs, negative bronchoalveolar lavage (BAL), and bronchoscopy cultures, as well as histological picture suggestive of drug-induced pneumonitis - diffuse alveolar damage and foamy macrophages in the alveoli in the absence of granulomas or giant cells - strongly favor the diagnosis of paclitaxel-induced pneumonitis [4]. In our case, CTPA revealed upper lobe mild subpleural reticular changes and mild mid-zone central ground-glass opacifications (Figure 1). PFTs demonstrated mild restrictive ventilatory defect and severe gas transfer defect (Table 1). Additionally, the patient’s condition continued to deteriorate despite treatment with azithromycin. These findings gave sufficient evidence to consider the temporal relationship between the patient’s first exposure to paclitaxel and the start of her symptoms. Hence, a diagnosis of PIP was made based on clinical presentation, radiographic pattern, exposure history, and exclusion of other causes of diffuse pulmonary infiltrates [6].

The main stay of management of PIP includes immediate cessation of paclitaxel and initiation of high-dose oral glucocorticoid within 24 hours of clinical diagnosis [1]. However, there is no established regimen and as such, systemic glucocorticoid therapy is based on the success of immunomodulation reported in previous case reports [4]. Of note, one study reported resolution of pneumonitis symptoms without the use of steroids in two cases [7]. In contrast to previous case reports, our patient was not placed on maintenance steroid therapy over months [1, 4, 6].

The consensus in the existing medical literature recommends against re-exposure to taxanes following a diagnosis of PIP in breast cancer patients [1, 4]. To the best of our knowledge, only two other studies have described a taxane rechallenge. In both case series, the patients met successful outcomes with no lung sequelae. The first case series described three patients in which paclitaxel was switched to an alternate agent, nanoparticle albumin-bound paclitaxel, known to be the least likely taxane to cause severe pneumonitis [8, 9]. However, a more recent case series of 19 patients described 17 patients being rechallenged with the same inciting taxane and two switched to docetaxel, similar to our patient [6]. Notably, our patient was not put on short-course oral steroids during and following the rechallenge for one week as a safety measure in contrast to the latter [6].

In our case, we considered the possibility of rechallenging with docetaxel due to our patient’s milder clinical course and complete clinical recovery. Chest X-ray revealed clear lungs. Moreover, PFTs demonstrated an improvement in DLCO (Hb) of 1.06 mL/min/mmHg i.e. from 50% to 60% predicted. A study found that affected patients had recovered clinically despite reduced carbon monoxide diffusion capacity on PFTs within six weeks of presentation [1]. Our patient was cleared to rechallenge with three cycles of docetaxel on the basis of clinical and radiological recovery. Following the first cycle, she did not experience respiratory deterioration. Subsequent PFTs demonstrated a decrease in DLCO (Hb) of 0.96 mL/min/mmHg i.e. from 60% to 55% predicted and an increase in DLCO/VA from 3.91 to 4.04 mL/min/mmHg/L. Despite reduced diffusion capacity, our patient was allowed to proceed with the remaining two cycles of docetaxel in the absence of pneumonitis symptoms. Following a diagnosis of PIP, the outcome of the docetaxel rechallenge was successful.

Our case report has some limitations due to a lack of certain tests being readily available. Our patient did not undergo BAL to exclude atypical infection. A leukocyte migration test was not performed to determine if paclitaxel caused the reaction. The diagnosis of paclitaxel-induced pneumonitis was not confirmed by lung biopsy, which would have excluded pulmonary infiltration with malignant cells. Additionally, a histologic picture of drug-induced pneumonitis combined with radiographic and PFTs findings would have further increased diagnostic accuracy. Subsequently, our patient was treated based on a clinical and radiological diagnosis.

## Conclusions

In conclusion, we report a case of PIP in Trinidad, West Indies, which has not previously been reported in this region. PIP should be suspected in any patient that presents with clinical respiratory symptoms while undergoing paclitaxel therapy, as this is a potentially fatal complication if left undiagnosed. Our patient illustrated clinical improvement of paclitaxel-induced pneumonitis following prompt discontinuation of paclitaxel and glucocorticoid therapy. Moreover, the patient had a favorable clinical outcome when rechallenged with docetaxel. We hope that this case report may lead to further research into docetaxel and other drugs being used as potential alternatives for patients diagnosed with PIP.
